# Peer Review of “Continuous User Experience Monitoring of a Patient-Completed Preoperative Assessment System in the United Kingdom: Cross-sectional Study”

**DOI:** 10.2196/35509

**Published:** 2022-01-06

**Authors:** Mathew Mbwogge

*This is a peer-review report submitted for the paper “Continuous User Experience Monitoring of a Patient-Completed Preoperative Assessment System in the United Kingdom: Cross-sectional Study.”*

## Round 1 Review

### General Comments

The need to enhance the quality of health care services and meet patient needs has prompted the development of applications that will improve patient flow and experience and cut back the cycle time during hospital visits. A review of telehealth interventions reported that such interventions can render the coordination of specialist services including surgery more efficient [1]. The extent to which the apps used in health care can be effective is dictated by the experiences of care users, who inevitably must be involved in the testing of these apps. This is because care user experience remains a major determinant of health care quality [2]. Common tools reported to be useful in measuring the usability of apps in mobile health interventions include the System Usability Scale (SUS), Health Information Technology Usability Evaluation Scale (ITUES), Post-Study System Usability Questionnaire (PSSUQ), Website Analysis and Measurement Inventory (WAMMI), and IBM Computer System Usability Questionnaire (CSUQ) [3].

In light of the above, the authors of the paper titled “Continuous user experience monitoring of a patient-completed preoperative assessment system: Usability evaluation and impact on completion times” [4] sought to investigate the usability of the MyPreOp app, factors affecting assessment questionnaire duration, and the effectiveness of a usability scale (the ITUES). They reported that while 80% of subjects had a good or better experience, 90% found the app easy to use based on the ITUES. The app’s usability was rated at 4.31, with a mean completion time of 46.95 (SD 25.83) minutes. The authors concluded that the user experience and usability of the app were high. Other studies have reported the testing of apps using other scales, with the most prominent being the SUS deployed in the testing of the “Be Prepared” app among subjects undergoing surgery [5], the “Patient Journey” app among subjects booked for surgery [6], and the “Pregnancy and Work” app among pregnant women [7] in the Netherlands, as well as the “mCare” app among subjects undergoing elective surgery in the United States [8] and a non-motor symptoms app among subjects with Parkinson disease in the United Kingdom [9].

Part of the impact of COVID-19 lies in the drive towards virtual care. The postpandemic era will demand more careful use of resources and better ways of improving patient experience. As such, this paper addresses a topic of growing interest in health care delivery and ties with the present global circumstances. The authors adhered to the IMRD standard of practice and the journal guidelines. The title throws an overall light on what the study is about but not how it was conducted. The Abstract is well structured and sums up the salient points of the paper but lacks the objectives. The Introduction and the Results are well presented, but the Methods and Discussion demand more attention, the improvement of which could affect other sections. The English used is plain language for easy understanding. That said, this paper could be improved further with the below recommendations:

### Specific Comments

1. The title of the paper needs formatting to conform to the journal guidelines.
2. Tables and figures need to be formatted according to the recommended standard.
3. The Abstract needs to conform to the BOMRC format as per the guidelines.
4. Authors need to reference specific guidelines used in reporting the results.
5. The methods of the study warrant improvement to make it robust and up to standard.
6. Some sections need to be moved and others reorganized for a better flow.
7. References could be improved further.

To elucidate the above specific comments, kindly refer to the below major and minor comments:

#### Major Comments

1. Kindly format your title following the guidelines [10]. A good title would be “Continuous User Experience Monitoring of a Patient-completed Preoperative Assessment System in The United Kingdom: Cross-sectional Study.”
2. I suggest improving the Methods subsection of the Abstract by also reporting (1) the study design, (2) setting and recruitment, (3) mean age and gender differences, (4) endpoints measured, (5) data collection methods, and (6) data analysis approach.
3. The below template may help in the overall structure of your paper: https://tinyurl.com/2p8c7yw6
4. Kindly structure your Introduction as follows:
  - Background (including the text on MyPreOp and the importance of usability)
  - Study rationale (why you thought the intervention would work, including similar studies)
  - Study aim and objectives
5. I do not understand the justification for placing the last paragraph of the Aim and Objectives subsection where it currently is. This should be moved to be part of your Rationale.
6. Your Methods section will be more robust if you could report according to:
  - Study design with justification (include studies that have used similar designs)
  - Study setting
  - Participant recruitment
  - Intervention and data collection (Phase 1 and Phase 2)
  - Endpoints measured (outcome and explanatory variables)
  - Study of the intervention (approach/measures used to assess that the outcome or observed impact was due to the intervention and not to other factors)
  - Data analysis (with justification for the approach used)
  - Ethical considerations (including ethical approval)
7. As part of your data analysis, kindly justify each analysis approach used, specifically with the usage of parametric and nonparametric tests.
8. Organize your *Data analysis* subsection (6.7 above) into:
  - Assessment of usability
  - Factors affecting questionnaire completion times
  - Effectiveness of the usability scale (also demonstrate the effect size using box plots)
9. Indicate the guidelines you used to report this study as part of your ethical considerations [11].
10. Kindly organize your *Results* section to follow your *Data analysis* subsection as follows:
  - Participant characteristics
  - Assessment of usability
  - Factors affecting questionnaire completion times
  - Effectiveness of usability scale
11. I suggest rephrasing your subtitle “Overall Data” as “Baseline” or “Participant Characteristics.”
12. There seems to be a mixup between parametric and nonparametric tests, as you talk of mean age and then the Wilcoxon rank-sum test. You report “In terms of mean age, participants in Phase 1 were younger than participants in Phase 2, and the difference was statistically significant (Wilcoxon sum rank test, *P*<0.001).” Could you please clarify? You also report “the mean scores for each question block were calculated and compared using the Kruskal-Wallis test.”
13. Based on (12) above and as reported and justified in your *Data analysis* subsection, I suggest adhering to a single statistical approach based on a normality test to report your results rather than using both parametric and nonparametric approaches, which may be confusing to readers. Report either means or medians in all your tables. You may include tables reporting both mean and median as Multimedia Appendices if very necessary.
14. Kindly correct from “Wilcoxon sum rank test” to Wilcoxon Rank Sum Test.
15. Regarding Table 3 under “Factors affecting completion times,” it will be good to announce whether the completion time was normally distributed and how this was verified. One may be tempted to ask why you used mean and not median completion times.
16. You may want to merge Tables 5, 6, and 7 as one table since all are based on a 4-point scale. I also suggest merging Tables 9 and 10 as one table.
17. It might be worthwhile to dedicate a paragraph at the end of your *Results* section to talking about contextual factors that intervened during the intervention and any unintended observed outcomes.
18. Kindly organize your Discussion into (1) Principal results, (2) Comparison with Prior studies, (3) Study limitations, and (4) Conclusion.
19. Move the text relating to ethical approval to the *Ethical Considerations* subsection in the *Methods* section.
20. Kindly list all Multimedia Appendices before the *References* section and move your list of abbreviations to the end of your paper, after the references.

#### Minor Comments

1. Maintain the corresponding author in the manuscript and add all others in the metadata section of the manuscript online management system.
2. Kindly include the objective subsection in your Abstract and state the study objectives.
3. The statement “So far, there have been no published studies that have investigated factors that influence the amount of time it takes for patients to self-complete a computerised preoperative assessment” appears too general. You may want to limit this to the United Kingdom and report it as “current evidence on the factors influencing...is limited.”
4. Kindly format your tables following the journal guidelines [12].
5. Do bear in mind that the maximum acceptable number of tables is 5. It is possible to merge some of the tables.
6. Kindly report all *P* values following the guidelines (eg, *P*<.001 and not *P*<0.001).
7. It is good to start your Conclusion with a statement of the study objectives. This should be followed by (1) a summary of findings, (2) lessons learned from your findings, (3) suggested direction of future research, and (4) recommendations.
8. Kindly replace your title “Declarations” with Acknowledgements. This should be followed by (1) Funding, (2) Author Contributions, and (3) Conflicts of Interest.
9. Your references need to be formatted following the journal guidelines. Set your reference manager to the AMA citation style and make sure to include a PubMed ID at the end of each reference. Include a DOI for all articles with a PMID and verify your DOIs using either https://www.doi.org/ or https://www.crossref.org to ensure they are working.
10. Make sure to trace the pdf version of articles that have neither a PMID nor DOI wherever possible.

## Round 2 Review

### General Comments

The authors of the paper titled “Continuous User Experience Monitoring of a Patient-completed Preoperative Assessment System in The United Kingdom: Cross-sectional Study” have done a great job in improving on the paper. However, I have 1 main concern regarding the present state of the paper.

### Specific Comments

The Discussion section needs further improvement.

#### Major Comments

I expect the length of your “Principal Results” in the *Discussion* section to be similar to the length of the first paragraph or at most the first two. May I please suggest that you use the text in the *Results* subsection of your Abstract as your “Principal Findings” and move any other text and discussion under “Principal Findings” to “Comparison with Prior Studies”?

## Abbreviations

CSUQ: Computer System Usability Questionnaire
ITUES: Information Technology Usability Evaluation Scale
PSSUQ: Post-Study System Usability Questionnaire
SUS: System Usability Scale
WAMMI: Website Analysis and Measurement Inventory
